# Aspirations to become an anaesthetist: longitudinal study of historical trends and trajectories of UK-qualified doctors’ early career choices and of factors that have influenced their choices

**DOI:** 10.1186/s12871-017-0392-5

**Published:** 2017-07-25

**Authors:** Beatrice Emmanouil, Michael J. Goldacre, Trevor W. Lambert

**Affiliations:** 1 UK Medical Careers Research Group, Unit of Health-Care Epidemiology, Nuffield Department of Population Health, University of Oxford, Oxford, UK; 2 Unit of Health-Care Epidemiology, Nuffield Department of Population Health, University of Oxford, Oxford, UK

**Keywords:** Anaesthesia, Workforce, Factors, Career choice, Gender differences

## Abstract

**Background:**

It is important to inform medical educators and workforce planners in Anaesthesia about early career choices for the specialty, factors that influence them and to elucidate how recent choices of men and women doctors relate to the overall historical trends in the specialty’s popularity.

**Methods:**

We analysed longitudinal data on career choice, based on self-completed questionnaires, from national year-of-qualification cohorts of UK-trained doctors from 1974 to 2012 surveyed one, three and 5 years post-qualification. Career destination data 10 years post-qualification were used for qualifiers between 1993 and 2002, to investigate the association between early choice and later destinations.

**Results:**

In years 1, 3 and 5 post-qualification, respectively, 59.9% (37,385), 64.6% (31,473), and 67.2% (24,971) of contactable doctors responded. There was an overall increase, from the early to the later cohorts, in the percentage of medical graduates who wished to enter anaesthesia: for instance year 1 choices rose from 4.6 to 9.4%, comparing the 1974 and 2012 cohorts. Men were more likely than women to express an early preference for a career in anaesthesia: for example, at year 3 after qualification anaesthesia was the choice of 10.1% of men and 7.9% of women. There was a striking increase in the certainty with which women chose anaesthesia as their future career specialty in recent compared to earlier cohorts, not reflected in any trends observed in men choosing anaesthesia.

Sixty percent of doctors who were anaesthetists, 10 years after qualifying, had specified anaesthesia as their preferred specialty when surveyed in year 1, 80% in year 3, and 92% in year 5.

Doctors working as anaesthetists were less likely than those working in other hospital specialties to have specified, as strong influences on specialty choice, ‘experience of the subject’ as students, ‘inclinations before medical school’, and ‘what I really want to do’. Men anaesthetists were more influenced in their specialty choice than men in other hospital specialties by ‘wanting a career with acceptable hours’; the corresponding difference among women was not significant.

**Conclusions:**

We suggest a focus on inspirational teaching of anaesthesia in medical school and on greater exposure to the specialty in the foundation programme. Factors which may discourage women from entering anaesthesia should be explored and addressed.

**Electronic supplementary material:**

The online version of this article (doi:10.1186/s12871-017-0392-5) contains supplementary material, which is available to authorized users.

## Background

Anaesthesia has been described as one of the most ‘holistic’ specialties in medicine, in the sense that it is involved in almost all clinical areas in hospital practice [1, 2]. It covers work in pre-, post- and intra-operative care in the majority of clinical procedural areas in hospital [3, 4], a burgeoning involvement in pre-hospital emergency and trauma care [5], as well as expertise in the management of critically ill patients and those with otherwise intractable acute and chronic pain. Anaesthesia and anaesthetists are therefore fundamental to an efficient and effective health service and it is important to consider questions of workforce supply and motivators for young doctors to give the specialty serious consideration as their career choice.

In the UK, although the popularity of anaesthesia as a specialty has increased over the last few decades [6, 7], there are concerns as to whether future numbers of doctors in anaesthesia will remain adequate in this consultant-led field [2, 8]. Rates of attrition in anaesthesia during the later stages of specialty training appear higher than in some other specialties [9].

Across the globe, there have been previous studies of early career choices and influences in anaesthesia but they have been based on small numbers [9–12]. The UK Medical Careers Research Group (UKMCRG) has been studying the career choices and future plans of doctors, nationally, factors that affect them, as well as eventual medical specialisation and career trajectories for over 40 years. In this study, by surveying all contactable doctors in the UK in years 1, 3 and 5 post-qualification, we have a unique database of information to inform trends over the years as well as to make comparisons of anaesthesia with other hospital specialties. Also, by consistently identifying important factors which affect doctors’ career preferences over four decades, we are able to give an overview of enduring traits which characterise anaesthesia choices as well as changes across generations in the career aspirations of men and women considering a career in anaesthesia.

Our study is set in the United Kingdom and is based on national cohort studies of UK medical graduates from the mid-1970s to the year 2016. We hope that its publication will interest anaesthetists and will prompt equivalent work in other countries. In particular, it would be helpful to identify whether young doctors in other countries who consider careers in anaesthesia have similar attributes to those in the UK.

In this study we were interested not only in choices and outcomes in anaesthesia careers, but also in identifying early career factors which had motivated those who eventually became anaesthetists. We have previously compared factors which motivated early career choices for anaesthesia as a future career to factors which motivated choices for other hospital specialties [6]. However, choice does not necessarily become reality in later years. Herein, we use the longitudinal nature of our study to relate early career choices for anaesthesia to eventual specialty destinations.

Our primary aim in this paper is to report the trends in early career choices for anaesthesia, and the relationship between choice and career outcome. Our secondary aim is to identify pertinent factors motivating career choices which were particularly important to those who became anaesthetists compared with doctors’ choices for other specialties.

## Methods

The UKMCRG has surveyed all doctors graduating from all UK medical schools in 1974, 1977, 1980, 1983, 1993, 1996, 1999, 2000, 2002, 2005, 2008, 2009, 2011 and 2012. With the exception of the 2009 and 2011 cohorts, which have only been surveyed once, and 1983 and 2012 which were only surveyed twice, all the cohorts were followed up at regular points, in years 1, 3, and 5 post-qualification. We also collected longer-term career data, reporting on graduates’ employment a decade after qualification, for five of these cohorts who graduated between 1993 and 2002.

Responses were collected by sending postal and, more recently, identical web survey questionnaires to the qualifiers, whose contact details were supplied by the UK General Medical Council. Up to four reminders were sent for each survey. Further details of the methodology are described elsewhere [10].

One, three and five years post-qualification, doctors were asked to specify up to three choices of eventual career specialty. They had the option to specify whether they had a single first choice (‘untied’), two equal first choices (tied) or whether all three choices (if three were given) were equally considered. Analysis for this paper focused on doctors’ choices for anaesthesia and intensive care/anaesthesia. Alongside the specification of eventual career choices, graduates were asked whether they had “not really”, “probably” or “definitely” made up their mind about their choice of career. Based on themes arising from existing literature as well those emerging from thematic analysis of previous responders’ comments, 12 factors potentially influencing career choices were identified and the doctors were asked to rate their influence as “not at all”, “a little” or “great deal” in year 1, 3 and 5.

Analysis of trends across different graduate cohorts and of differences in time passing from qualification (1, 3, or 5 years) is purely chronological to ensure exactitude and for ease of comparability with studies that may be done elsewhere. We do not relate results to specific timings in the training of anaesthetists because training pathways tend to be revised at regular intervals and the timings of key training events (such as postgraduate examinations) do not necessarily remain constant over time. Details of recent career pathways in the UK for all doctors [11] and for anaesthesia specifically [12] as well as a brief historical perspective on training in the UK are described elsewhere [13].

SPSS 22 was used for all statistical analyses. We report standard summary statistics throughout, with statistical significance of inferences set to *p* < .05. We used χ^2^ statistics (reporting Yates’ continuity correction when there was one degree of freedom) to compare distributions of responses. When comparing multiple distributions, we used z-tests with Bonferroni corrections. Trends were investigated using Mantel-Haenszel linear-by-linear χ^2^ tests of association.

## Results

### Response rates

Fourteen cohorts of UK doctors, between 1974 and 2012, were surveyed 1 year post-qualification and the overall response rate was 59.9% (37,385 out of 62,464). Across the 12 cohorts surveyed in year 3, 64.6% (31,473 out of 48,702) responded. Across the ten cohorts surveyed in year 5, the response rate was 67.2% (24,971 out of 37,162). Response rates were calculated after excluding doctors who had previously declined participation, were deceased or were untraceable.

### Career choice

**Fig. 1:**
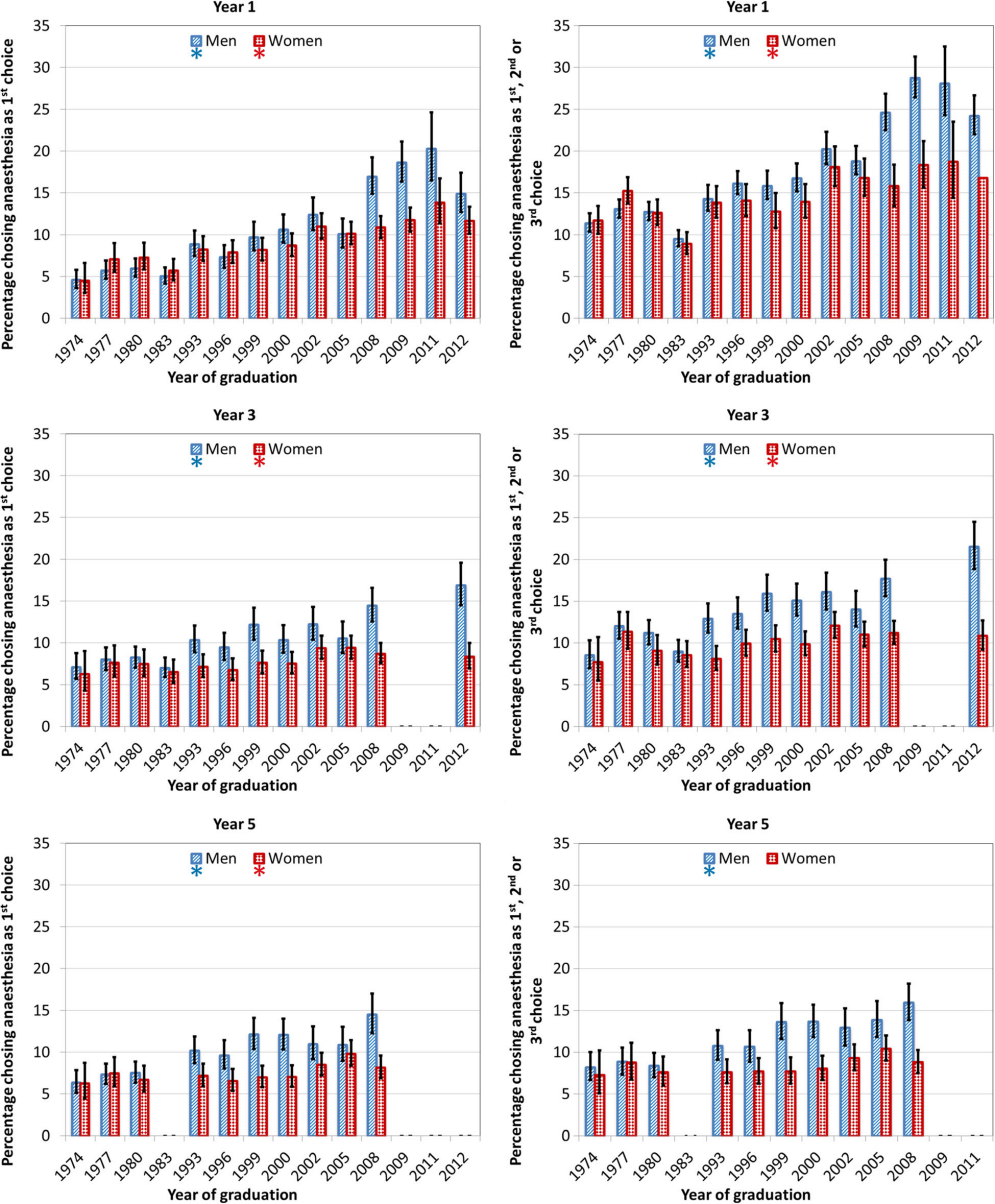
Percentages of men and women doctors whose first choice (*left*) or any of three choices (*right*) was for anaesthesia in years 1, 3 or 5 by cohort. *denotes a statistically significant linear trend across cohorts

Figure 1 shows the percentages, with 95% confidence intervals, of men and women who chose anaesthesia in each individual graduation cohort from 1974 to 2012, as first choice and as any of three specialty choices. The numbers on which Fig. 1 is based are tabulated in Additional file 1.

For cohorts graduating from 1974 to 2012, anaesthesia was the first choice of 9.4% of doctors and the first, second or third choice of 15.8%, in year 1 after graduation. Whilst there was no significant difference between the percentages of men (9.5%) and women (9.4%) whose first choice was anaesthesia in year 1, χ^2^ = 0.1, *p* = .75, significantly more men (16.6%) than women (15.1%) chose anaesthesia as any of three choices (χ^2^ = 15.9, *p* < .001). Anaesthesia has become more popular in recent years; there was a significant linear increase across the cohorts from 1974 to 2012 (Fig. 1) in the proportion of both men and women choosing anaesthesia as first choice (men χ^2^ = 325.0, women χ^2^ = 84.1, *p* < .001 for both) or any choice (men χ^2^ = 294.1, women χ^2^ = 53.9, *p* < .001 for both).

In year 3, a higher proportion of men (10.1%) than women (7.9%) chose anaesthesia as their preferred first choice of future career (χ^2^ = 45.6, *p* < .001) and as any choice (men 13.4%, women 10.2%, χ^2^ = 76.6, *p* < .001). There was an upward trend in choosing anaesthesia from 1974 to 2012 for men (first choice: χ^2^ = 96.6, any choice: χ^2^ = 116.6, *p* < .001 for both) and women (first choice: χ^2^ = 10.0, any choice: χ^2^ = 9.7, *p* = .002 for both). Overall, 9% of all doctors in year 3 chose it as a first choice and 11.8% as any choice.

Gender differences continued in year 5, with anaesthesia being chosen by a higher percentage of men than women, both as first choice (9.7% of men, 7.6%, of women, χ^2^ = 35.9, *p* < .001) and as any choice (11.2% of men and 8.4% of women, χ^2^ = 53.9, *p* < .001). There was an upward linear trend across cohorts in the percentages of male doctors choosing anaesthesia in year 5 as a first choice (χ^2^ = 68.4, *p* < .001) but for females the statistical evidence for a trend was weaker (χ^2^ = 6.21, *p* = .01). For anaesthesia as any choice, the upward trend only applied to men (χ^2^ = 294.1, *p* < .001) with the result for females being non-significant (χ^2^ = 3.7, *p* = .054). Across all the cohorts surveyed at year 5, 2159 doctors (8.6%) chose anaesthesia as their first choice of career in year 5 whilst 2441 (9.8%) put anaesthesia down as any of three preferred careers.

### Certainty of choice

The confidence with which doctors chose anaesthesia as their future career specialty in early years after qualification was also examined, looking at historical trends over the years as well as comparing men and women choosing anaesthesia to doctors in other hospital specialties. In detail, we asked doctors to score whether they had “definitely”, “probably” or “not really” made up their mind about their choice of career specialty, and compared the responses of those who chose anaesthesia to all other hospital specialties (combined) as their first choice of career.

**Fig. 2:**
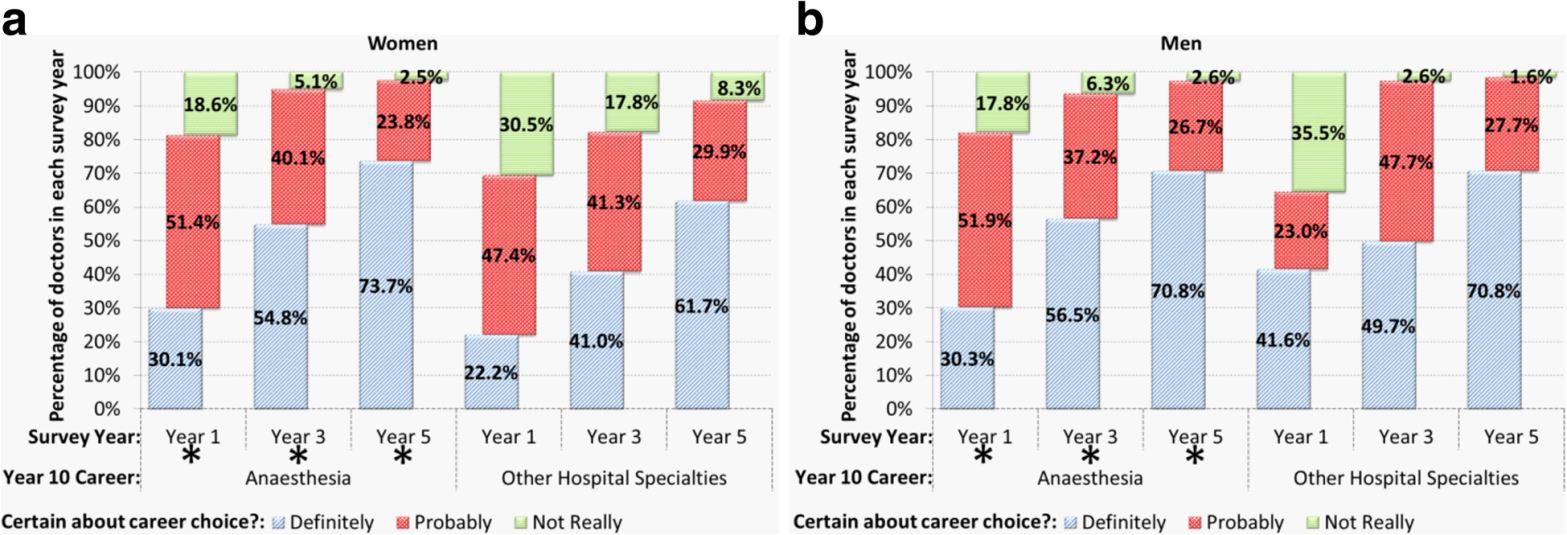
Percentages of **a** women and **b** men doctors by untied first choice in years one, three and five post-qualification who responded that they were “definitely”, “probably” or “not really” certain about their choice of long-term career. *denotes a statistically significant difference in comparing percentages of doctors in anaesthesia and those in other hospital specialties

Overall, doctors who chose anaesthesia as their first choice were significantly more certain about their choice than doctors who chose other hospital specialties (combined). In years 1, 3, and 5 respectively, 30, 56, and 72% of respondents who chose anaesthesia were definite about their choice compared with 24, 42 and 64% of those who chose other hospital specialties. The level of certainty of choice depended on the specialty chosen, in years 1, 3, and 5 (χ^2^(2) = 111.91, 245.84, and 77.50 respectively, all *p* < .001). Women choosing anaesthesia were more certain of their choice than women who chose other hospital specialties (*p* < .05) in years 1, 3 and 5 (Fig. 2). Among male doctors the picture was more complex: men who chose anaesthesia in year 1 were less sure of their choice than men who chose other hospital specialties (*p* < .05); by year 3 this was reversed (*p* < .05); and by year 5 the level of certainty comparing choices for anaesthetics and for other hospital specialties was very similar (*p* > .05).

Combining the cohorts, gender differences in the levels of certainty of doctors who chose anaesthesia were small in all three survey years (Fig. 2). However, the historical trends across the cohort years differed for female and male doctors. In particular, there was a striking increase in the certainty with which women chose anaesthesia in recent cohorts. Women’s definiteness about their choice of anaesthesia rose from 15 to 49% in year 1, from 48 to 67% in year 3, and from 52 to 81% in year 5, comparing the 1974 cohort with the latest cohort surveyed in each year (2012 in years 1 and 3, 2008 in year 5). A chi square test of linear trend across the cohorts was significant in each year: χ^2^ = 26.3, 9.6, 12.8 respectively for years 1,3,5; all *p* < .001). For men the increase in certainty in year 1 was much smaller across the cohorts, for example in year 1 it rose from 42.0% of 1974 graduates to 47.3% of 2012 graduates. There was no evident linear trend in any year-from-qualification: χ^2^ = 4.8, 1.96, 2.3 respectively for years 1,3,5; *p* = .28, .16, .13).

### Choice and destinations

The importance of early specialty choices of doctors was investigated by matching early choices with their career destination in year 10 after qualifying (Table 1). Of all doctors who were anaesthetists 10 years after qualifying, 60% had chosen this specialty as an untied first choice in year 1, 80% in year 3, and 92% in year 5. Other combinations of ‘matching’ between early choices and later destinations are shown in Table 1. Men were slightly more likely than women to match on early choices and later destinations; but differences between them were generally small and not statistically significant.

**Table 1:** Percentages (numbers /row-total) of doctors working in anaesthesia 10 years after qualification within each original choice group in years 1, 3 and 5

	Doctors working as anaesthetists in year 10		
Original Choice of Anaesthesia	All	Women	Men
Untied first choice Year 1	60.3% (374/620)	56.8% (172/303)	63.7% (202/317)
Tied first choice Year 1	51.7% (445/860)	46.8% (205/438)	56.9% (240/422)
Not chosen in year 1	3.1% (238/7599)	2.5% (108/4254)	3.9% (130/3345)
Untied first choice Year 3	79.5% (578/727)	76.9% (256/333)	81.7% (322/394)
Tied first choice Year 3	74.5% (627/842)	72.3% (279/386)	76.3% (348/456)
Not chosen in year 3	1.4% (115/8004)	1.1% (48/4483)	1.9% (67/3521)
Untied first choice Year 5	91.5% (664/726)	90.1% (302/335)	92.6% (362/391)
Tied first choice Year 5	88.6% (738/833)	87.6% (332/379)	89.4% (406/454)
Not chosen in year 5	0.5% (45/8219)	0.4% (17/4628)	0.8% (28/3591)

We also studied consistency of choice. Of doctors in anaesthesia in year 10, 43% had told us in all of years 1, 3 and 5 that they wanted to be anaesthetists; 25% had told us this was their untied first choice in years 3 and 5 but not 1; and 8% had chosen specialties other than anaesthetics as their untied first choice in years 1, 3 and 5. Other combinations of choice are shown in Table 2. Table 2 also shows the early career choices for anaesthesia – few in number – of doctors who were in hospital specialties other than anaesthesia in year 10.

**Table 2:** Percentage of doctors working in anaesthesia in year 10, tabulated by their career preferences in combinations of years 1, 3 and 5; and percentages of doctors in other hospital specialties in year 10 tabulated by their early preferences for anaesthesia

Specialty in year 10						
	Anaesthesia			Other hospital specialties		
When did they choose Anaesthesia as their only first choice?	ALL	Women	Men	ALL	Women	Men
	*N* = 608	*N* = 281	*N* = 327	*N* = 6518	*N* = 3769	*N* = 2749
Year 1, Year 3 and Year 5	42.8%	45.6%	40.4%	0.2%	0.2%	0.3%
Years 3 and 5	25.2%	24.9%	25.4%	0.2%	0.2%	0.2%
Year 5 only	11.5%	13.5%	9.8%	0.2%	0.2%	0.3%
Years 1 and 5	3.3%	1.8%	4.6%	0.0%	0.0%	0.1%
Never as unique first choice	8.1%	7.5%	8.6%	96.0%	96.2%	95.8%
Years 1 and 3	3.6%	2.5%	4.6%	0.5%	0.4%	0.3%
Year 3 only	4.1%	3.6%	4.6%	0.7%	0.7%	0.6%
Year 1 only	1.5%	0.7%	2.1%	2.1%	2.1%	2.2%

### Factors influencing early career choice

The influence on career choice of each of 13 factors was examined, comparing the responses of anaesthetists with those working in other hospital specialties. Having established that the great majority of doctors who were anaesthetists at year 10 had chosen anaesthesia in year 5, we report on their responses in year 5 in Table 3. Equivalent data from the doctors’ replies in years 1 and 3 are shown in Additional file 2. Table 3 describes the proportion of doctors in each career group rating each factor as having “a great deal” of influence on their career choice. “Enthusiasm/commitment to the specialty: what I really want to do” was the factor scored most highly by both anaesthetists and doctors in other specialties. Anaesthetists were less likely than other hospital specialists to specify, as strong influences on specialty choice, experience of the subject as students, inclinations before medical school, and ‘what I really want to do’. Compared with other hospital specialty doctors, men (but not women) anaesthetists were more influenced by ‘wanting a career with acceptable hours’. For women, the only factor considered influential by significantly more anaesthetists than doctors in other hospital specialties was ‘career and promotion prospects’.

**Table 3:** Factors influencing specialty choice a great deal, as specified by the doctors in year 5: 1.) Comparison of doctors in anaesthesia and in other hospital specialties in year 10. 2.) Comparison of men and women working in anaesthesia in year 10 (gender comparison column). Percentages in bold denote a statistically significant difference (χ^2^ with Bonferroni corrections) between the distributions in anaesthesia and those in other careers within each gender for a particular factor

Percentages rating factor as influencing career choices “A great deal”							
Factor	Men working in			Women working in			Gender comparison
	Anaesthesia	Other hospital specialties	*p*	Anaesthesia	Other hospital specialties	*p*	*p*
Wanting a career that fits my domestic situation	22.3	22.2	1	33.5	32.7	.82	**.001**
Wanting a career that fits with acceptable hours/ working conditions	**42.1**	**35.4**	**.01**	51.3	47.8	.25	**.01**
Future financial prospects	10.7	14.3	.06	5.6	4.5	.41	**.02**
Career and promotion prospects	32.9	29	.12	**30.4**	**19.7**	**<.001**	.53
Self-appraisal of own skills/ aptitudes	61	57.8	.24	62.9	60.4	.41	.65
Advice from others	15.3	15	.93	16	13	.14	.87
Experience of chosen subject as a student	**16.7**	**25.4**	**<.001**	**16.6**	**27.3**	**<.001**	1.00
A particular teacher / department	**18.1**	**30.3**	**<.001**	**17.6**	**30**	**<.001**	.98
Inclinations before medical school	**4.8**	**12.8**	**<.001**	**2.4**	**10.7**	**<.001**	.12
Experience of job so far	72	71	.73	73.9	71.6	.41	.61
Enthusiasm / commitment: what I really want to do	**71.7**	**80.1**	**<.001**	**72.7**	**81.7**	**<.001**	.82
Other reasons	29.3	33.3	.53	38.8	33	.43	.28
Financial circumstances whilst training	3.3	5.9	.36	1.3	3.6	1	.68

We also compared the influences on career choice of men and women anaesthetists. Women anaesthetists were more motivated than men to seek a career that framed ‘lifestyle’ factors, with a higher proportion of women than men citing seeking a career that suited their domestic situation and acceptable working hours. Conversely, boosting financial prospects was an influencing factor for a higher percentage of men anaesthetists than women.

## Discussion

### Main findings

In recent decades, there has been a steady rise in the numbers and percentages of both male and female doctors who expressed a career preference for anaesthesia in years one, three and five after qualification. In the most recent cohorts, around 10% of doctors expressed a career preference for it. As context, in the 2000s in England, about 7% of all senior doctors (consultants and principals in general practice) were anaesthetists [14].

In all cohorts, and at all career stages, a higher proportion of men than women were attracted to anaesthesia. It is important to determine whether there are any remediable factors that deter women from opting for a career in anaesthesia.

Women’s confidence in choosing anaesthesia, in the early years after qualification, has increased substantially in the last 40 years and, overall, women who do choose anaesthesia now seem more certain about their specialty choice than women who chose other hospital specialties. These trends were reflected in men but to a smaller degree. Unsurprisingly, confidence in their specialty choices increased for both sexes after year 3. The majority of eventual anaesthetists (60%) had expressed a preference for this specialty in year 1 and almost all had done so in year 5 (92%). Expectedly, choosing anaesthesia consistently from year 1 to year 5 was a good predictor of future career destinations. Anaesthetists had been less influenced in their specialty choice than other hospital doctors by intentions prior to medical school, by experience of the specialty in medical school, and by experience of the specialty as doctors. There may be a case for a focus on inspirational teaching of anaesthesia in medical school and on greater exposure to the specialty in the foundation programme (i.e. in the early years of general postgraduate medical training). There were two noteworthy differences between men and women in factors influencing career choice that differentiated anaesthetists from other hospital doctors. Men working in anaesthesia had been significantly more interested in working conditions than men in other hospital specialties; for women this was not a distinguishing factor for anaesthetists. Also, a higher percentage of women anaesthetists than women in other specialties rated opportunities for career progression highly, whereas all men doctors rated this factor comparably. Gender comparisons amongst anaesthetists revealed that a higher percentage of women than men anaesthetists rated wanting acceptable hours and working conditions, and a career that fits with their domestic situation, as important and fewer women than men were motivated by financial prospects.

### Comparison with existing literature

The proportions of doctors choosing anaesthesia reported here are in line with proportions of doctors choosing anaesthesia when surveyed immediately after qualification recently in the UK (12%) [7, 15] and in New Zealand (15%) [16] where anaesthesia was more popular with men than women. Career choices of doctors with some rotational anaesthesia experience in Pakistan [17] echoed the gender differences (15% men and 8% women chose anaesthesia). In Rwanda, 8% of doctors in year 3 post-qualification chose anaesthesia either as first, second or third choice; however in Nigeria only 0.7% of doctors overall chose anaesthesia.

We also extend previous associations between early choices and later career [6] by determining that by year 5 the majority of anaesthetists will have either conclusively entered the speciality or quit, if they started in it. Whilst anaesthesia is a popular choice and all initial training posts in the UK are filled [2, 7], there have been difficulties in retention of trainees in later specialist training stages [9], which indicates that any attrition takes place predominantly before year 5. Some doctors who initially expressed a preference for anaesthesia may not have been working in it at year 10 because they changed their minds, or did not secure training posts, or did not progress in the specialty (or maybe did not continue in medicine at all).

In a recent study in Scotland, 42 doctors at several stages of anaesthesia training retrospectively rated the influence of factors on their career choice [9]. Whilst difficult to compare directly, there are parallel themes with our current findings, with career prospects and hours of work being more important than financial prospects. Interestingly, financial prospects were important to people who did not choose anaesthesia in Rwanda, where wages for anaesthetists were quite modest and there was no prospect of augmenting them with private work [18], whilst in India anaesthetists cited financial motivations as positively influential in their choice [19]. The relatively smaller influence of learning environment for anaesthetists compared to other specialty doctors might indicate that there is room for improvement. In Nigeria, poor teaching was a factor in not choosing anaesthesia [20]. Finally, gratification in, and commitment to aspects of the nature of anaesthesia as a specialty, emerged as a reason for selecting anaesthesia in US resident doctors, reflecting our current findings [21].

### Strengths and limitations

This study has the advantage of being the largest and longest running cohort study of anaesthetists’ careers in the UK. It offers insights into choices at different career stages and factors that affect them, general trends over 40 years, and comparisons with other hospital specialities to draw conclusions on the motivational differences in the making of anaesthetists. However, surveying doctors at regular time intervals rather than key training junctures limits study of possible associations of career choices and factors with exact training stages. Also, more detailed questionnaires, delving into the anaesthesia learning environment, [22] may reveal specialty-intrinsic factors that attract or deter people to and from anaesthesia and suggest further policy improvements at different stages in anaesthesia education.

### Implications

Doctors who became anaesthetists appear to have been less influenced in their career decisions by factors related to their learning environment or early inclination, than were doctors who became specialists in other areas of hospital medicine. One might therefore inform policies by revising curricula to include innovative learning such as serious games [23] in anaesthesia at earlier stages as well as re-examining selection processes to attract doctors who will become dedicated practitioners of anaesthesia. In examining factors attracting women doctors in anaesthesia from other hospital specialties we found that women anaesthetists were positively influenced by career and promotion prospects in the specialty, more so than women who pursued other careers. Both men and women anaesthetists scored highly on wanting a career with acceptable hours and working conditions. This may indicate areas for policy initiative when publicising the attractions of careers in anaesthesia to prospective trainees. To expand the pool of candidates, it is important to address the lower percentage of women than men who choose anaesthesia, in the context of the current gender profile of medical graduates in which women are in the majority.

## Conclusions

We report a continuous, four-decade-long, rise in the percentages of both male and female doctors who prefer anaesthesia as their future career. In spite of women’s dramatic increase in confidence when choosing anaesthesia in more recent cohorts, the field remains more popular amongst men than women - reflecting world-wide reports in relative gender analyses. By examining trajectories of early career preferences and associating them with year 10 career destinations we deduce that attrition takes place predominantly within the first 5 years post-qualification. The information that attrition takes place during earlier post-graduate training years, combined with the findings that doctors who are more influenced by teaching are more likely to end up working in other hospital specialties, begs the examination of training practices and an increased focus on inspirational teaching. Capitalising on the career influences that apparently attracted doctors to anaesthesia rather than to other hospital specialties, policy initiatives could focus on maintaining and promoting clear career promotion pathways and working conditions with optimal working hours.

## Additional files

Additional file 1: A table of Numbers (percentages) of doctors whose first choice (a) or any of three choices (b) was anaesthesia by cohort for years one, three and five post qualification. (DOCX 28 kb)

Additional file 2: A table of Factors influencing specialty choice a great deal, as specified by the doctors in a year 1 and year 3. (DOCX 27 kb)
