# Supplementary material for: Aspirations to become an anaesthetist: longitudinal study of historical trends and trajectories of UK-qualified doctors’ early career choices and of factors that have influenced their choices
Source: BMC Anesthesiol. 2017 Jul 25;17:100. doi: 10.1186/s12871-017-0392-5 (PMC5526313; doi:10.1186/s12871-017-0392-5)
Supplement: Supplementary file 1 — A table of Numbers (percentages) of doctors whose first choice (a) or any of three choices (b) was anaesthesia by cohort for years one, three and five post qualification. (DOCX 28 kb) [file 12871_2017_392_MOESM1_ESM.docx]

Table of Numbers (percentages) of doctors whose first choice (a) or any of three choices (b) was anaesthesia by cohort for years one, three and five post qualification.

| a) | **Any first choice is Anaesthesia N (%)** | | | | | | | | | | |
| --- | --- | --- | --- | --- | --- | --- | --- | --- | --- | --- | --- |
|  | **Year One** | | | **Year Three** | | | | **Year Five** | | | |
| **Cohort** | **Men** | **Women** | **Total** | **Men** | **Women** | **Total** | | **Men** | **Women** | | **Total** |
| 1974 | 65 (4.6%) | 24 (4.5%) | 89 (4.6%) | 76 (7.1%) | 26 (6.3%) | 102 (6.9%) | | 80 (6.4%) | 31 (6.3%) | | 111 (6.3%) |
| 1977 | 101 (5.7%) | 62 (7.1%) | 163 (6.2%) | 124 (8.0%) | 60 (7.6%) | 184 (7.9%) | | 126 (7.3%) | 66 (7.5%) | | 192 (7.4%) |
| 1980 | 110 (6.0%) | 74 (7.3%) | 184 (6.4%) | 150 (8.2%) | 76 (7.5%) | 226 (7.9%) | | 130 (7.5%) | 66 (6.7%) | | 196 (7.2%) |
| 1983 | 96 (5.0%) | 72 (5.7%) | 168 (5.3%) | 127 (7.0%) | 79 (6.5%) | 206 (6.8%) | | N/A | N/A | | N/A |
| 1993 | 115 (8.9%) | 109 (8.2%) | 224 (8.5%) | 144 (10.4%) | 99 (7.1%) | 243 (8.8%) | | 137 (10.2%) | 99 (7.2%) | | 236 (8.6%) |
| 1996 | 102 (7.3%) | 121 (7.9%) | 223 (7.6%) | 120 (9.5%) | 98 (6.7%) | 218 (8.0%) | | 112 (9.6%) | 89 (6.6%) | | 201 (8.0%) |
| 1999 | 113 (9.7%) | 128 (8.2%) | 241 (8.8%) | 134 (12.2%) | 110 (7.6%) | 244 (9.6%) | | 140 (12.1%) | 105 (7.0%) | | 245 (9.2%) |
| 2000 | 139 (10.6%) | 146 (8.7%) | 285 (9.6%) | 138 (10.4%) | 123 (7.5%) | 261 (8.8%) | | 143 (12.1%) | 107 (7.1%) | | 250 (9.2%) |
| 2002 | 135 (12.4%) | 185 (11.0%) | 320 (11.5%) | 130 (12.2%) | 158 (9.4%) | 288 (10.5%) | | 108 (11.0%) | 133 (8.5%) | | 241 (9.4%) |
| 2005 | 119 (10.1%) | 197 (10.1%) | 316 (10.1%) | 106 (10.5%) | 160 (9.4%) | 266 (9.8%) | | 98 (10.9%) | 143 (9.8%) | | 241 (10.2%) |
| 2008 | 193 (17.0%) | 235 (10.9%) | 428 (13.0%) | 171 (14.5%) | 178 (8.7%) | 349 (10.8%) | | 121 (14.5%) | 125 (8.1%) | | 246 (10.4%) |
| 2009 | 186 (18.6%) | 225 (11.7%) | 411 (14.1%) | N/A | N/A | N/A | | N/A | N/A | | N/A |
| 2011 | 75 (20.3%) | 87 (13.8%) | 162 (16.2%) | N/A | N/A | N/A | | N/A | N/A | | N/A |
| 2012 | 133 (14.9%) | 177 (11.6%) | 310 (12.9%) | 139 (16.9%) | 104 (8.3%) | 243 (11.7%) | | N/A | N/A | | N/A |
| **Total** | **1682 (9.5%)** | **1842 (9.4%)** | **3524 (9.4%)** | **1559 (10.1%)** | **1271 (7.9%)** | **2830 (9.0%)** | | **1195 (9.7%)** | **964 (7.6%)** | | **2159 (8.6%)** |
| b) | **Any choice is Anaesthesia N (%)** | | | | | | | | | | |
|  | **Year One** | | | **Year Three** | | | | **Year Five** | | | |
| **Cohort** | **Men** | **Women** | **Total** | **Men** | **Women** | | **Total** | **Men** | **Women** | **Total** | |
| 1974 | 160 (11.3%) | 62 (11.7%) | 222 (11.4%) | 91 (8.5%) | 32 (7.7%) | | 123 (8.3%) | 103 (8.2%) | 36 (7.3%) | 139 (7.9%) | |
| 1977 | 230 (13.0%) | 133 (15.2%) | 363 (13.8%) | 186 (12.0%) | 89 (11.3%) | | 275 (11.8%) | 152 (8.8%) | 77 (8.7%) | 229 (8.8%) | |
| 1980 | 234 (12.7%) | 128 (12.6%) | 362 (12.7%) | 204 (11.2%) | 92 (9.0%) | | 296 (10.4%) | 145 (8.4%) | 75 (7.6%) | 220 (8.1%) | |
| 1983 | 181 (9.5%) | 113 (8.9%) | 294 (9.3%) | 163 (9.0%) | 104 (8.5%) | | 267 (8.8%) | N/A | N/A | N/A | |
| 1993 | 185 (14.3%) | 183 (13.8%) | 368 (14.0%) | 179 (12.9%) | 112 (8.1%) | | 291 (10.5%) | 145 (10.8%) | 105 (7.6%) | 250 (9.2%) | |
| 1996 | 225 (16.1%) | 215 (14.1%) | 440 (15.0%) | 171 (13.5%) | 144 (9.9%) | | 315 (11.6%) | 124 (10.6%) | 104 (7.7%) | 228 (9.0%) | |
| 1999 | 184 (15.8%) | 200 (12.8%) | 384 (14.1%) | 175 (15.9%) | 151 (10.4%) | | 326 (12.8%) | 157 (13.6%) | 116 (7.7%) | 273 (10.3%) | |
| 2000 | 219 (16.8%) | 233 (13.9%) | 452 (15.2%) | 201 (15.1%) | 161 (9.8%) | | 362 (12.2%) | 162 (13.7%) | 122 (8.0%) | 284 (10.5%) | |
| 2002 | 221 (20.3%) | 305 (18.1%) | 526 (18.9%) | 171 (16.1%) | 203 (12.0%) | | 374 (13.6%) | 127 (12.9%) | 146 (9.3%) | 273 (10.7%) | |
| 2005 | 222 (18.8%) | 327 (16.8%) | 549 (17.6%) | 141 (14.0%) | 187 (11.0%) | | 328 (12.1%) | 125 (13.8%) | 152 (10.4%) | 277 (11.7%) | |
| 2008 | 280 (24.6%) | 342 (15.8%) | 622 (18.8%) | 209 (17.7%) | 229 (11.2%) | | 438 (13.6%) | 133 (15.9%) | 135 (8.8%) | 268 (11.3%) | |
| 2009 | 287 (28.8%) | 352 (18.3%) | 639 (21.9%) | N/A | N/A | | N/A | N/A | N/A | N/A | |
| 2011 | 104 (28.1%) | 118 (18.7%) | 222 (22.2%) | N/A | N/A | | N/A | N/A | N/A | N/A | |
| 2012 | 216 (24.2%) | 255 (16.8%) | 471 (19.5%) | 177 (21.5%) | 135 (10.8%) | | 312 (15.1%) | N/A | N/A | N/A | |
| **Total** | **2948 (16.6%)** | **2966 (15.1%)** | **5914 (15.8%)** | **2068 (13.4%)** | **1639 (10.2%)** | | **3707 (11.8%)** | **1373 (11.2%)** | **1068 (8.4%)** | **2441 (9.8%)** | |
