# Supplementary material for: Aspirations to become an anaesthetist: longitudinal study of historical trends and trajectories of UK-qualified doctors’ early career choices and of factors that have influenced their choices
Source: BMC Anesthesiol. 2017 Jul 25;17:100. doi: 10.1186/s12871-017-0392-5 (PMC5526313; doi:10.1186/s12871-017-0392-5)
Supplement: Supplementary file 2 — A table of Factors influencing specialty choice a great deal, as specified by the doctors in a year 1 and year 3. (DOCX 27 kb) [file 12871_2017_392_MOESM2_ESM.docx]

Table of Factors influencing specialty choice a great deal, as specified by the doctors in a ) year 1 and b) year 3: comparison of doctors in anaesthesia and in other hospital specialties in year 10. Percentages in bold denote a statistically significant difference (χ^2^ with Bonferroni corrections) between the distributions in anaesthesia and those in other careers within each gender for a particular factor.

| 1. Percentages rating factor as influencing year 1 choices “ A great deal” | | | | |
| --- | --- | --- | --- | --- |
| Factor | **Men working in** | | **Women working in** | |
|  | **Anaesthesia** | **Other hospital specialties** | **Anaesthesia** | **Other hospital specialties** |
| Wanting a career that fits my domestic situation | 8.6 | 9.6 | 16.2 | 14.6 |
| Wanting a career that fits with acceptable hours/ working conditions | **38.9** | **27.7** | **46.7** | **34.4** |
| Future financial prospects | 16.5 | 16.6 | 10.1 | 6.7 |
| Career and promotion prospects | **32.3** | **26.4** | **30.8** | **20.4** |
| Self-appraisal of own skills/ aptitudes | 48.5 | 49.7 | 46.2 | 51.3 |
| Advice from others | 18.3 | 15.8 | 18.3 | 16.5 |
| Experience of chosen subject as a student | **39.2** | **48.6** | **37.9** | **50.4** |
| A particular teacher / department | **21.6** | **31.9** | 29 | 32.7 |
| Inclinations before medical school | **5.8** | **15.9** | **5.2** | **16.2** |
| Experience of job so far | 48.1 | 52.7 | 49.1 | 55.3 |
| Enthusiasm / commitment: what I really want to do | **52.6** | **68.8** | **60.2** | **69.5** |
| Other reasons | 27.8 | 37.7 | 35.3 | 39.9 |
| Financial circumstances whilst training | 8.6 | 9.6 | 16.2 | 14.6 |
| 1. Percentages rating factor as influencing year 3 choices “ A great deal” | | | | |
| Factor | **Men working in** | | **Women working in** | |
|  | **Anaesthesia** | **Other hospital specialties** | **Anaesthesia** | **Other hospital specialties** |
| Wanting a career that fits my domestic situation | 9.3 | 13.8 | 12.8 | 19.2 |
| Wanting a career that fits with acceptable hours/ working conditions | **46.9** | **29.9** | **50.7** | **39.5** |
| Future financial prospects | 16.7 | 14.7 | 9.6 | 6.4 |
| Career and promotion prospects | **37.8** | **25.4** | **29.8** | **19.3** |
| Self-appraisal of own skills/ aptitudes | 50.4 | 53.7 | 47.5 | 53 |
| Advice from others | 20.3 | 15.3 | **24.7** | **16.1** |
| Experience of chosen subject as a student | 24.2 | 26.3 | **17.8** | **32.8** |
| A particular teacher / department | **19.6** | **32.8** | 22.9 | 32.2 |
| Inclinations before medical school | **6.7** | **14.6** | **1.6** | **13.2** |
| Experience of job so far | 60.3 | 67.6 | 67 | 72 |
| Enthusiasm / commitment: what I really want to do | 57.5 | **67.1** | 64.4 | 70.6 |
| Other reasons | 19 | 32.6 | 33.3 | 40.2 |
| Financial circumstances whilst training | 4.8 | 5.6 | 6.2 | 5.8 |
